# Pushing partnerships: corporate influence on research and policy via the International Life Sciences Institute

**DOI:** 10.1017/S1368980019005184

**Published:** 2020-08

**Authors:** Sarah Steele, Gary Ruskin, David Stuckler

**Affiliations:** 1 Department of Politics and International Studies, University of Cambridge, Cambridge, UK; 2 Intellectual Forum, Jesus College, Cambridge, UK; 3 U.S. Right to Know, Oakland, CA, USA; 4 Dondena Research Centre and Department of Policy Analysis and Public Management, University of Bocconi, Milano, Italy

**Keywords:** Industry, Research, Corporate influence, International Life Sciences Institute

## Abstract

**Objective:**

There are concerns that some non-profit organisations, financed by the food industry, promote industry positions in research and policy materials. Using Freedom of Information (FOI) requests, we test the proposition that the International Life Sciences Institute (ILSI), one prominent non-for profit in international health and nutrition research, promotes industry positions.

**Design:**

U.S. Right to Know filed five FOI from 2015 to 2018 covering communications with researchers at four US institutions: Texas A&M, University of Illinois, University of Colorado and North Carolina State University. It received 15 078 pages, which were uploaded to the University of California San Francisco’s Industry Documents Library. We searched the Library exploring it thematically for instances of: (1) funding research activity that supports industry interests; (2) publishing and promoting industry-sponsored positions or literature; (3) disseminating favourable material to decision makers and the public and (4) suppressing views that do not support industry.

**Results:**

Available emails confirmed that ILSI’s funding by corporate entities leads to industry influence over some of ILSI activities. Emails reveal a pattern of activity in which ILSI sought to exploit the credibility of scientists and academics to bolster industry positions and promote industry-devised content in its meetings, journal and other activities. ILSI also actively seeks to marginalise unfavourable positions.

**Conclusions:**

We conclude that undue influence of industry through third-party entities like ILSI requires enhanced management of conflicts of interest by researchers. We call for ILSI to be recognised as a private sector entity rather than an independent scientific non-profit, to allow for more appropriate appraisal of its outputs and those it funds.

Multi-national food, beverage, agrichemical and supplements industries fund significant research and activities around public health and nutrition. It is widely acknowledged that researchers’ and professionals’ ties to industry have the potential to influence their objectivity, enabling industry-friendly research and messages to reach the public in ways that veil any potential influence^(1)^. Although major multinational companies including Mars Inc. and The Coca-Cola Company (hereafter Coke) have recently committed to improving the transparency of their research and professional activities following increasing press scrutiny^(2)^, declarations by those they fund in journals and scientific circulars are not always complete^(3)^, often providing little or inadequate information about the involvement of funding entities and their industry sponsors^(4,5)^.

Further complicating the picture, multinational companies often distribute funds via intermediary bodies, such as non-profit organisations that support research, engagement and policy activity favourable to the industry’s interests, like the International Food Information Council (IFIC)^(6)^ and the now closed Global Energy Balance Network (GEBN)^(3)^. When these organisations are cited as funders, often there is a lack of understanding that they receive vast amounts of support from industry, despite them having been found by researchers and the press to be lobbying and fronting industry interests^(7–10)^.

One major industry-funded institution active in public health and nutrition is the International Life Sciences Institute (ILSI), a non-profit established in 1978 by Dr. Alex Malaspina. Malaspina was previously a Coke Scientific and Regulatory Affairs executive, who founded ILSI and then was its President until 2001. He has maintained involvement in ILSI’s conferences and funding activities, including awarding the Malaspina International Scholars Travel Award with ILSI and Coke, long after his retirement however^(11–13)^. ILSI is registered as a non-profit [501(c)(3)] and states its mission as being to ‘provide science that improves human health and well-being and safeguards the environment’^(14,15)^.

At present, ILSI comprises eighteen bodies designed to promote a ‘global partnerships for a healthier world’, which include: the ILSI Research Foundation, ILSI Health and Environmental Institute, ILSI Europe, ILSI North America, ILSI Mexico, ILSI Mesoamerica, ILSI North Andean, ILSI Brazil, ILSI Argentina, ILSI South Andean, ILSI Middle East, ILSI South Africa, ILSI Southeast Asia Region, ILSI Taiwan, ILSI Korea, ILSI India, ILSI Focal Point in China and ILSI Japan^(16)^. These bodies are brought together through a strategy called ‘One ILSI’^(16)^. ILSI received an initial endowment of $22 million from the industry^(11)^, expanding globally over the following decades^(14)^, and its bodies are now funded primarily by industry ‘members’, with ILSI reporting that of its US$17.7 million income in 2015, $11·6 million came from what was termed ‘member support’ (see Box 1)^(17)^.

Box 1: Sources funding to ILSI

ILSI acknowledges it receives funding from industry. The documents we received reveal that in 2012, ILSI received $528 500 in contributions from CropLife International, a $500 000 contribution from Monsanto and $163 500 from Coca-Cola^(18)^.

A draft 2013 ILSI Internal Revenue Service tax return shows that ILSI received $337 000 from Coca-Cola and more than $100 000 each from Monsanto, Syngenta, Dow AgroSciences, Pioneer Hi-Bred, Bayer Crop Science and BASF^(19)^.

A draft 2016 ILSI North America Internal Revenue Service tax return shows a $317 827 contribution from PepsiCo, contributions greater than $200 000 from Mars, Coca-Cola and Mondelez and contributions greater than $100 000 from General Mills, Nestle, Kellogg, Hershey, Kraft, Dr. Pepper Snapple Group, Starbucks Coffee, Cargill, Unilever and Campbell Soup^(20)^.

ILSI maintains that despite this funding, it is neutral and ‘is a non-profit, worldwide organization whose mission is to provide science that improves human health and well-being and safeguards the environment. ILSI and ILSI entities foster public-private scientific partnerships to address knowledge gaps to benefit public good’^(21)^.

Despite claims of independence, ILSI’s bodies have been subject to scrutiny for their corporate engagements with questions about whether ILSI – which we refer to here as all eighteen of its organisational bodies – acts to front for the food and beverage industries, promoting the commercial interests of their members, or these industry on the whole, around the world, including in emerging markets like China^(22,23)^. In 2000, even those at the WHO have questioned its nature and engagements with different industries, including the tobacco industry^(24)^. Mars Inc. pulled out of its ILSI membership, effective from the end of 2018, with public statements that ILSI is involved in ‘advocacy-led studies’, implicitly outing it as a front group, following public controversy over a research study that ILSI had backed^(25)^. This following a process of the global candy maker committing to more robust transparency and openness, a trend emerging in the face of increasing public scrutiny over the commercial determinants of health. Notably, Mars’ statements supported previous anecdotal accusations around ILSI’s involvements, implying that it likely was deploying tactics and learning from tobacco’s past strategies to thwart regulation, thus reopening discussion about whether indeed ILSI is a non-profit acting for the public good or indeed an arm of private entities acting to further commercial interests under another face.

In the wake of these public accusations, we ask: how much separation there really is between industry and researchers when industry-funded groups like ILSI provide the backing for a study? Does ILSI promote industry positions? We use US state Freedom of Information (FOI) requests entered into the University of California San Francisco Library’s Industry Documents Library to explore how ILSI conducts itself with researchers working in North America and what is said about the organisation and its former and present executives about ILSI North America, ILSI Global and ILSI Research’s activities and conduct.

## Methods

Between June 2015 and February 2018, U.S. Right to Know, a non-profit consumer and public health group, submitted US state public records requests – henceforth referred to as FOI – to public academic institutions: Texas A&M, the University of Illinois, University of Colorado and North Carolina State University. U.S. Right to Know subsequently sent these requests to the UCSF database, which are now available at https://www.industrydocuments.ucsf.edu/food/. U.S. Right to Know has extensive experience in transparency research and selected the relevant persons to request information from due to their known past connections with industry or industry-funded bodies. The requests yielded a total of 15 078 pages in PDF format, including 1347 pages from Texas A&M, 346 pages from the University of Illinois, 11 714 pages from the first FOI to the University of Colorado and a further 221 pages on the second FOI to it, and 1450 pages from North Carolina State University. An independent researcher (S.S.) explored the documents in the database for indications of ILSI’s activities and role.

These documents were analysed by two members of the research team, who read the received documents independently of each other. One researcher (G.R.) undertook an initial review of the documents received by manually reading all the received pages of the documents, searching for interactions between ILSI bodies and corporations or individual names known to be associated with industry members, including Alex Malaspina, leading to a follow-up FOI with the University of Colorado due to his on-going engagement with ILSI across the initial document batch period, despite ILSI’s claims to the contrary recently on its website^(21)^. Because these requests capture diverse communications, and often involved someone from ILSI being cc’d into an email on an unrelated subject, documents that made no reference to ILSI’s activities directly were not tagged by this researcher.

The second researcher (S.S.), trained in law and public health, then went through the database to identify emails or attachments containing information on ILSI and industry interaction, analysing them for instances of potential industry influence, as well as seeking to highlight instances where data disconfirmed – that is, conflicted with, refuted or provided a negative instance of – the themes and categories of analysis^(26)^. She close read all results in the database pertaining to ILSI and extracted relevant results to for discussions of ILSI by its employees or others who either work with it now, of have done so in the past, either as employees or as trusted others. We provide references to ILSI’s own statements, policies and practices to allow readers to compare the email text to their own organisational positions. We also note the position of those who sent the emails in relation to the organisation and note where the information is historical and therefore may not be current practice. Our research therefore sits as a case study, shedding light on industry influence and pointing out potential issues needing further investigation. The emails act to highlight potential sites for further engagement and research on improving practice.

To do this, the researcher (S.S.) engaged in a descriptive qualitative process of data analysis, which aims to explore a set of related but distinct categories of industry manipulation and influence, searching for instances that confirm or refute views that ILSI acts in this manner. She studied the FOI emails and all received attachments, which included some meeting minutes, to identify instances of commonality and variation with those types in White and Bero’s framework, working with those categories that could be applied to food entities and non-profits^(27)^. The researcher coded instances of discussion of: (1) funding research that supports industry interests; (2) publishing industry-sponsored positions or literature under ILSI without reference to industry; (3) dissemination of favourable material to decision makers and/or the public through ILSI or ILSI organised events and (4) suppression of views that do not support industry through ILSI. We note that this is not a hierarchical arrangement; the categories in a typology are related and not subsidiary to one another.

Following best qualitative practices, to the extent possible, we quote directly from the documents ‘in their own words’. To reduce bias, all researchers convened to discuss the identified content and consider alternative interpretations. We report all emails referenced in an online accessible format, so that our interpretations are accessible and replicable to all readers and the public. It was not possible to perform quantitative analysis of the received emails without comprehensive fully representative sample of the universe of possible quotes, since the batch is likely incomplete. We looked qualitatively for instances that confirmed or refuted an instance, rather than measuring numerical frequency.

## Results

In line with White and Bero’s framework^(27)^, we identified four forms of industry involvement through ILSI’s activity globally. We consider each in turn.

### International Life Sciences Institute funding research activity that supports industry interests

ILSI funds extensive research and other activities, including meetings and conferences, internationally through its constituent bodies. Attachments to the emails reveal ILSI supports travel and attendance at these conferences and professional meetings, providing allowances for those promoting research or favourable positions to policy makers, professional associations and scientific meetings^(28)^. ILSI cites that these are reimbursements for expenses^(21)^. We note, however, that such reimbursements should be declared generally as past research on the pharmaceutical industry suggests travel payments, meeting attendance costs reimbursements, food and beverage provision, and the like can influence professional decision-making and practices^(29,30)^. This research confirms that it is not simply grants, employment or large gifts that have influence but also smaller activities that can create a favourable predisposition that might consciously or subconsciously impact on research.

We also note the emails confirm that corporations can earmark contributions to support specific ILSI initiatives. For example, the Coca-Cola Company gave $325 000 for 2014 and $350 000 for 2015 to fund Platform for International Partnerships activities (ILSI’s programme for ‘managing ILSI’s interactions’ with the WHO and FAO) and the Malaspina International Scholar Travel Award (which is ILSI’s outreach programme for young scientists)^(28)^. Draft minutes from a 2015 ILSI Board of Trustees meeting reveal that several of its programmes are funded specifically by The Coca-Cola Company, which directs how these funds are used:

The Restricted Programs include the ILSI Platform for International Partnerships and the contributions from The Coca-Cola Company. The latter have been distributed as requested by the donor to specific activities, e.g., Malaspina International Scholars Travel Award, and food safety training in Asia and in Africa. The Branch Staff Travel grant fund is also included in the Restricted Programs. The International Brach (sic) Activity includes funding held for the ILSI Focal Point in China (operating funds as well as training funds) and Latin American branches coordination. A new, part-time Latin American branches coordinator position has been established with funding from The Coca-Cola Company^(28)^.

While it is not unusual for donors to earmark funds for a specified purpose, the completeness of disclosures around the sources of ILSI funding of research varies significantly among publications it supports. For example, in one recent study that concluded controversially that ‘there seems to be no reliable evidence indicating that any of the recommended daily caloric thresholds for sugar intake are strongly associated with negative health effects’, the funding statement made no specific mention of industry involvement, instead only listing funding by ILSI. Following publication of an Associated Press article questioning it^(31)^, along with subsequent expression of concerns, a correction was issued^(32)^ and the statement updated to read:

Financial Support: This project was funded by the Technical Committee on Dietary Carbohydrates of ILSI North America. The authors wrote the protocol, the scope of which was reviewed and approved by ILSI and conducted the study independently from ILSI^(32)^.

Subsequently, new, more robust examples have emerged around ILSI funding:

The present review results from a workshop organized by the European Branch of ILSI Europe. This publication was coordinated by Dr. Peter Putz, Scientific Project Manager at ILSI Europe. The workshop was funded by the ILSI Europe Obesity and Diabetes Task Force, the ILSI Europe Metabolic Imprinting Task Force, ILSI Brazil, ILSI North America and ILSI Southeast Asia Region. Industry members of the taskforces are listed on the ILSI Europe website at http://www.ilsi.eu. For further information about ILSI Europe, please email info@ilsieurope.be or call +32 2 771 00 14. The opinions expressed herein and the conclusions of this publication are those of the authors and do not necessarily represent the views of ILSI Europe nor those of its member companies^(33)^.

This example was mirrored in other recent publications^(34)^, and we note that declaring affiliations, any research support and funding, including of travel allowances and stipends, are critical to allow the reader a clearer picture of any potential bias, whether conscious or unconscious.

We also note that ILSI that it is ‘well aware of potential conflict of interest issues and has been actively studying and publishing on this topic’, with a series of recent papers considering scientific integrity and openness^(35–38)^.

### Publishing industry-approved positions or literature under International Life Sciences Institute without reference to industry input

The emails also revealed that since ILSI is aware that industry ties may reduce the credibility of its papers, any influence on papers by industry should not extend to co-authorship. An email exchange between Dr. Bruce Chassy, Professor Emeritus of the Department of Food Sciences and Human Nutrition at the University of Illinois, and Dr. Kevin Glenn, Monsanto Senior Science Fellow and the Scientific Affairs Pipeline Lead within Monsanto Regulatory and past Chair of the International Food Biotechnology Committee of ILSI, highlights the use of academic authors to add authority, while allowing industry hidden input into ILSI publications^(39)^. Chassy writes:

… I think to have credibility the article should be authored by academics. That is not to say anybody who wants to help shouldn’t be able to contribute useful parts to it, but it needs to be distanced from industry. I have shared my view with both you and Ray before that having industry and academics co-write papers for ILSI could come back to bite all of us some day. Not a good idea. A minor niggle is that while some industry people like you and Ray write very well, in general academics are better and faster at it. That said a manuscript produced by a committee will always be a bit of a camel^(39)^.

In response Glenn writes:

Your comments about ILSI and academic/industry co-authoring are a great summary of yesterday’s IFBiC [ILSI International Food Biotechnology Committee] meeting – we have (I guess you would say “finally”) seen the light and will be implementing an advisory group that is predominantly populated by academic and non-industry scientists (to provide overall strategic direction) – and for future task force efforts, to have academic/regulatory scientists be the “Core” – that meets initially with a broad tri-partite group – but then completes the work on their own – bringing it back to the group not for “approval” but for a final reality check (or whatever). … And the authorship of the “unintended effects” paper (if it materializes) – would be academic and non-industry – I feel I am mainly coordinating, at best…^(39)^.

What becomes clear from this exchange is the input that industry partners and ILSI itself have to articles that list academic and non-industry authorships listed. Initiating projects through a ‘tri-partite group’ – with representatives from academia, industry and government – which then delegates this to academics who later bring academic work back for industry and ILSI’s ‘final reality check’ illustrates the scope for influence^(39)^. This aligns with ILSI’s belief that ‘it is in the public interest to bring together scientists from industry, academia and government to address scientific issues of public health concern’^(21)^.

Notably, such an exchange validates Mars Inc.’s acknowledgement that two of its employees were cc’d into emails about a research project leading to the controversial *Annals of Internal Medicine* article discussed above, which led Mars to state publicly that it would communicate to ILSI the inappropriateness of such emails, while emphasising that ‘the paper undermines the work of public health officials and makes all industry-funded research look bad…’^(40)^. Mars Inc. labelled this as ‘advocacy’ for industry – its own language – although ILSI maintains publicly that it ‘explicitly prohibits its member entities from ‘advocat[ing] the commercial interests of their member companies or other parties’’^(21)^.

### Disseminating favourable material to decision makers and/or the public through International Life Sciences Institute or International Life Sciences Institute’s organised events with academics

Publicly, ILSI states that it ‘does not lobby, nor does ILSI seek to influence individuals, positions, and/or specific policy’^(21)^. Rather, ILSI maintains that it ‘brings forward precompetitive science that *informs* actions by industry, government, academia, and/or other researchers’^(21)^. Indeed, ILSI maintains that it partners with different entities, and the Platform for International Partnership has long formed part of ILSI’s core strategy to influence at the international level or as ILSI’s own meeting minutes phrase it, to manage ‘ILSI’s interactions with WHO and FAO’^(41)^.

We note that in board meeting minutes obtained via FOI requests, amendments were proposed (and then passed) to ILSI’s bylaws to ensure that its status as an organisation in official relations with the WHO could be maintained. This followed from concerns by the WHO about ILSI India’s ties to a company with connections to the tobacco industry, leading to a proposal to remove ILSI’s status. The minutes make clear that through the process, ILSI’s status as a non-governmental organisation was being called into question, as the WHO had come to the conclusion that ILSI should be regarded as a ‘private sector entity’. As the minutes detailed, the WHO Executive Board has four categories – (1) non-governmental organisations, (2) private sector entities, (3) philanthropic foundations and (4) academic institutions – and ILSI categorised itself as a scientific/academic entity in an online questionnaire distributed by WHO^(42)^. Rather notably, ILSI was willing to forgo recognition at the WHO if it listed ILSI as a ‘private sector entity’. The minutes state: ‘…[s]hould WHO insist on calling ILSI a private sector entity, ILSI may have to end all activities with WHO to avoid such a classification’^(42)^. The minutes highlight that the WHO views ILSI differently than ILSI regards itself. ILSI contests its view, we suggest, because if it was considered a private body, this could threaten both ILSI’s regulatory and tax situation in the US, where its operation centre, and because this would amount to recognition that ILSI is an extension of industry rather than a publicly beneficial non-profit organisation as ILSI maintains publicly. The WHO declined to renew ILSI’s special status in 2017 in any event.

Certainly, the public–private partnerships’ approach guides ILSI to engage with policy makers and national organisations that do lobby. We found email correspondence from 2015 that reveal how ILSI-produced guidelines on public–private partnerships were promoted at a National Academy of Sciences meeting. In its Symposium Session proposal, ILSI sets out the aim of the session, to address:

… the evolving role of industry funding in nutrition research and explore existing public-private partnerships that are leveraging available resources to make advances in nutrition science^(42)^.

In a reply email to Malaspina, Suzanne ‘Suzie’ Harris, then-Executive Director of ILSI, answers his enquiry about the adoption of ILSI’s guidelines by saying:

The meeting yesterday was very successful. Four professional societies – ASN, IFT, AND (new name for the ADA) and IAFP (International Association for Food Protection) – all agreed to endorse the public-private partnership approach and will so state in upcoming journal editorials. Dr. Michael McGinnis encouraged these societies to work to remove barriers to having everyone work together to solve public health problems. So a real feather in Eric’s cap!^(42)^

This perspective is further promoted on the Oxford University Press page for ILSI’s journal, *Nutrition Reviews* ^(43)^. The emails reveal that ILSI uses its platforms to promote this industry being at the table in discussions, research and meetings, in effect advocating for industry engagement. ILSI suggests that this is so as to ‘address topics of common interest’ and that ‘its unique public-private structure … fills knowledge gaps and serves society in ways that any one entity on its own cannot. ILSI does not lobby, conduct lobbying activities, or make policy recommendations’^(44)^.

But does ILSI enforce these policies? ILSI published a press release in 2015, published on its website in 2019, that details sanctions that it placed on ILSI-Mexico for engaging in lobbying behaviour^(45)^. However, other email exchanges we see involve Malaspina in June 2015 engaged in an email chain, which includes Suzie Harris, then-Executive Director of ILSI, concerning then-WHO Director General Margaret Chan, who had raised questions about sugar-sweetened products. In these emails he states:

We must find a way of some one [SIC] such as a famous scientist arrange to pay her a visit. Jim Hill or some one [SIC] of similar stature or a US Government scientist. As the President of ILSI I had a special and productive luncheon with the former DG, Dr Nakajime in 1995 at his private dining room in the WHO Geneva Headquarters to tell him about ILSI and how the two organisations could work with each other. In 1999 I visited the new DG Mrs Brutland in Geneva, when I invited her, on behalf of the World Economic Forum, to come to the Davos meeting of 1999, and be the Keynote Speaker at the Food Governors special dinner… By the way, the future Coke President, Mr Neville Isdell attended that dinner with me. In summary I am suggesting that collectively we must find a way to start a dialogue with Dr Chen. If not, she will continue to blast us with significant negative consequences on a global basis. This threat to our business is serious^(40)^.

What we see here is historic contextual information from the Founder of ILSI, suggesting that prior to his retirement in 2001, the organisation lobbied international leaders regularly, and he proposes a similar approach now. We note that Malaspina, while no longer leading ILSI, still continues to represent the organisation at events, conferences and on its social media, showing his continued engagement and influence. It is clear that the organisation’s own founder sees its role as one of lobbying and advocacy, suggesting further investigation is warranted. We also found statements reporting that ILSI works with other bodies like the IFIC to disseminate information favourable to industry, stating that ‘ILSI generates the scientific facts and IFIC communicates them to the media and public’^(46)^. This suggests a need to explore how ILSI is extending its influence and authority through public relations and science communication and not just research.

### Suppression of views that do not support industry

As the emails about Margaret Chan imply, Malaspina holds the view that ILSI has a critical role in seeking to dismiss views that were unhelpful to industry and ILSI’s activities. The emails reveal active targeting of those who raise questions about the health risks of processed food ingredients and products. In particular, ILSI has been active in convening those who question the role of sugar-sweetened beverages in the global obesity epidemic and challenging individuals who advocate restrictions on sugar-rich beverage consumption. In the email chain about Chan, Malaspina then asks Dr Barbara Bowman, then CDC’s Director of the Division for Heart Disease and Stroke Prevention:

Any ideas how we can have a conversation with WHO? Now, they do not want to work with industry. Who finds all the new drugs. Not WHO, but industry. She is influenced by the Chinese Govt and is against US. Something Must be done [SIC].^(40)^

Bowman then proceeds to provide advice on whom to approach to influence the WHO on sugar and beverage policy matters and promotes ILSI’s central role, stating:

Am wondering whether anyone with ILSI China, perhaps Madame Chen, might have ideas. Another thought, perhaps someone with connections to the PEPFAR program. Or Gates and Bloomberg people, many have close connections with the WHO regional offices. …^(40)^

Notably, when Bowman’s interactions with Malaspina were publicised^(47)^, Bowman stepped down from her CDC role and subsequently two members of US Congress requested investigation by the Inspector General of the Department of Health and Human Services^(48)^. Critically, what we also see here is that Malaspina sees ILSI has involved in targeting of individuals who promote perspectives that could harm corporate interests. It confirms the involvement that ILSI has had historically, not only in disseminating food industry narratives through persons like James O. Hill, then a prominent researcher at University of Colorado Denver and member of the National Academy of Medicine, now on ILSI-NA’s Board of Trustees and Department Chair at the University of Alabama, Birmingham, who was CC’d into the email, but also in working to co-opt and convert dissenters through targeted approaches.

Indeed, the email exchanges suggest that ILSI has played a key defensive role for the food industry when its interests are challenged. In an email from Michael Ernest Knowles, a former Coca-Cola VP and ILSI president, to Malaspina, Knowles lays out his views on how the food industry should respond to challenges regarding the health risks of its products. Knowles recounts the need for:

the generation of credible, consensus science on the issues hitting the industry – obesity and causative factors, sugar, low/no calories sweetener safety – in particular we need to use external organizations …^(49)^

The first organisation Knowles mentions that industry should use for such product defence is ILSI. He notes that:

these issues need to be addressed now in the traditional manner of ILSI – in a transparent manner with the best international experts and the full proceedings published and further publicized by IFIC^(49)^.

The emails continue with Malaspina explaining the urgency of responding to the new dietary recommendations. This view is confirmed by Dr. James Hill, Director of the Center for Human Nutrition at the University of Colorado Health Sciences Center, who states in an email to Malaspina that:

…GEBN and ILSI could be, in my opinion, synergistic in addressing this issue.

Frankly, we need the food industry to step up to provide more resources both to ILSI and GEBN. When things like this happen, individual companies tend to want to keep their head down. If they do this, our opponents will win and we will all lose.

I do not think we can overestimate the importance of dealing aggressively with this issue.

Jim^(50)^.

GEBN was the Global Energy Balance Network, an organisation established by Coca-Cola which promoted a narrative that minimised the role of sugar-sweetened beverages in the obesity epidemic, which has since been shuttered^(51)^. We suggest that these emails convey that ILSI acts on behalf of industry to deploy international experts to defend industry positions where unfavourable legislation, policy or guidance is being pursued at national, regional or international bodies, or by professional societies and groups.

## Discussion

The emails we received and analysed thematically reveal that various influential individuals who have held leadership roles at ILSI historically or at present suggest that ILSI promotes industry positions and influence while purporting publicly to be neutral and guided by science. While ILSI-funded research is becoming more transparent in terms of declarations of ILSI’s industry ties, there are many examples where industry links to research are obscured. We are especially concerned by emails suggesting that ILSI can be deployed to marginalise unfavourable positions, which supports the argument that it is a front for industry when positions need to be quashed.

However, our research is subject to limitations. First, FOI requests can, at best, provide only a partial picture and there is an inevitable risk of bias in sampling and interpretation. Second, some documents may have been deleted, destroyed or withheld from us, so we do not claim the sample is comprehensive. Third, following best practices to mitigate any potential analytical bias, we have reported text ‘in their own words’, with verbatim copies, and provide all cited emails in appendices. This allows reader to make their own interpretations as well as to replicate our interpretations. Fourth, our research focused on ILSI, one prominent international nutrition non-profit. We are unable to ascertain the extent to which ILSI’s activities are reflective of a more general pattern among corporate funded non-profits. Finally, although we have been limited to studying a set of those emails that have been released, which unlike the tobacco documents archive cannot be exhaustive, they are sufficient to reveal positive evidence that ILSI has acted in ways which undermine its claims to be a scientifically objective organisation. Within the emails we received, we sought actively to disconfirm these findings in the email document set (e.g. to look for instances that refuted our findings) and were unable to do so. Taken together, the available evidence is consistent with an alternative proposition that ILSI acts to promote the corporate agenda of its funders.

Without transparency and openness measures being widely in place, industry interests can direct how research funds routed through non-profit bodies are generally allocated and what topics and findings will be supported for publication. With regard to ILSI, when Mars Inc. withdrew its support, its public statements raised concern about potential corporate influence on the research which ILSI funds. In its departure statement, the Company states that ‘[w]e do not want to be involved in advocacy-led studies that so often, and mostly for the right reasons, have been criticized…’^(25)^. The Company’s statements followed criticism that research by ILSI was being used to cast doubt on the influence of sugar on overweight and obesity, discussed below, and that disclosures in scientific publications were problematically incomplete about what ILSI funding actually involved.

These findings are troubling as previous research on studies of sugar-sweetened beverages identified how those with food industry funding were five times more likely to report no positive association between weight gain and obesity than those not reporting funding^(1)^. Industry-funded reviews are more likely to suggest that evidence for a causal relation between sugar-sweetened beverages and weight gain was weak^(52)^. While ILSI-funded studies are increasingly noting connections between it and industry, not all declarations are complete. ILSI does, however, suggest it is conducting research into improving conflicts of interest issues and has recently restructured and issued new governance directives^(21)^. We hope that this indicates a move forward from the behaviour discussed in the emails we received.

However, ILSI still engages in the conception, funding and promotion of research, and it funds researchers to travel to its meetings and to events run by other bodies, while facilitating partnering between public and private entities^(21)^. Reimbursements of expenses are routinely provided, according to ILSI itself^(21)^. Between Mars Inc. suggestion of advocacy and work being viewed in a ‘bad’ light^(2)^, and past research which suggests even benefits not just funding can influence favourable messaging^(29)^, there is cause for concern and on-going follow-up research. The continued pushing of a partnership model should be further explored in light of ILSI’s recent reforms.

Given the WHO position and the issues raised by former member Mars Inc., we call for ILSI and other similar industry-backed non-profits, which are in effect bodies that are an extension of these industry actors, to be recognised as private sector actors, and for their claims of scientific neutrality to be subject to intense scrutiny by those engaging with the content that they produce. We also argue that conflict of interest statements must become more robust when such bodies provide funding, detailing how those bodies are themselves funded and what non-grant support and reimbursements have also been provided in the past and present studies. Where scientists, academics and government officials receive support channelled by industry through ILSI, such as travel awards and conference expenses, this too should be acknowledged both in peer-reviewed and non-peer-reviewed content. Currently, the failure to include robust declarations on all outputs means the media and others risk promoting industry-sponsored research as independent and objective without an ability to judge any potential bias and influence.

Additional research is needed to ensure that journals with links to such organisations are truly independent, with robust peer-review and editorial processes, or whether industry influence extends into editorial decision-making. It is still unclear whether ILSI’s advocacy extends to the content of the research it funds and its journal and publications. Further research is needed.
